# Suicidal ideation and attempt among school going adolescents in Bhutan – a secondary analysis of a global school-based student health survey in Bhutan 2016

**DOI:** 10.1186/s12889-019-7791-0

**Published:** 2019-12-02

**Authors:** Tashi Dema, Jaya Prasad Tripathy, Sangay Thinley, Manju Rani, Tshering Dhendup, Chinmay Laxmeshwar, Karma Tenzin, Mongal Singh Gurung, Tashi Tshering, Dil Kumar Subba, Tashi Penjore, Karma Lhazeen

**Affiliations:** 1 HMIS Evaluation and Research Section, Policy and Planning Division, Ministry of Health, Thimphu, Bhutan; 2 International Union Against Tuberculosis and Lung Disease, Paris, France; 3 Department of Community Medicine, All India Institute of Medical Sciences, Nagpur, India; 4 School Health Programme, Department of Public Health, Ministry of Health, Thimphu, Bhutan; 5 Regional Office for South East Asia, World Health Organization, New Delhi, India; 6 Policy and Planning Division, Ministry of Health, Thimphu, Bhutan; 7 Médecins Sans Frontières, New Delhi, India; 8 Khesar Gyalpo University of Medical Sciences of Bhutan, Thimphu, Bhutan; 9 Mental Health Programme, Department of Public Health, Ministry of Health, Thimphu, Bhutan; 10 Department of Public Health, Ministry of Health, Thimphu, Bhutan

**Keywords:** Suicidal behaviour, GSHS, Adolescent, Sexual violence, Parental engagement

## Abstract

**Background:**

Suicide is one of the leading causes of death and Disability Adjusted Life Years (DALYs) worldwide. The economic, emotional and human cost of suicidal behaviour to individuals, families, communities and society makes it a serious public health issue. We aim to determine the prevalence and factors associated with self-reported suicidal behaviour (suicidal ideation and attempt) among school going adolescents (13–17 years).

**Methods:**

This is a secondary analysis of a nationally representative data for Bhutan namely Global School Based Student Health Survey in 2016 which reports on various dimensions of adolescent health including suicidal behaviour. The survey employed a multistage sampling method to recruit participants aged 13–17 years (*n* = 5809) from 50 schools (25 each in rural and urban area). The survey used an anonymous self-administered pre-tested 84-item questionnaire. Weighted analysis was done. Adjusted prevalence ratios (aPRs) and adjusted Odds Ratios (aORs) have been presented with 95% confidence intervals (95% CI).

**Results:**

A total of 667 (11.6%) adolescents reported considering a suicide attempt whereas 656 (11.3%) reported attempting suicide in the past 12 months. Among those reporting suicidal ideation, 388 (58.6%) reported attempting a suicide and 274 (41.4%) had ideation alone, whereas, 247 (38.9%) reported attempting a suicide without previous ideation. Female sex, food insecurity, physical attack, sexual violence, bullying, feeling of loneliness, low parental engagement, reported worry about lack of sleep, urge to use drugs/alcohol, smokeless tobacco use, drug abuse and parental smoking were the factors associated with suicidal attempt. All these factors except smokeless tobacco use and parental smoking were associated with suicidal ideation. Having helpful/close friends was found to be protective against suicide ideation.

**Conclusion:**

Suicidal behaviour among school going adolescents in Bhutan is high and alarming, especially among girls. Bullying, sexual violence, feeling of loneliness and drug abuse were some of the key risk factors identified. It is important to identify these risk factors early and effectively tackle them in order to prevent suicides. It requires a multi-faceted intervention with the support of the children, community, teachers and parents.

## Introduction

Suicide is one of the leading causes of death and Disability Adjusted Life Years (DALYs) worldwide. The economic, emotional and human cost of suicidal behaviour to individuals, families, communities and society makes it a serious public health issue. Nearly 800,000 people die due to suicides and many more attempt suicide every year [1, 2]. Suicide is the second leading cause of death among 15–29 year olds. Around 78% of all suicides occurred in low- and middle-income countries (LMICs) in 2015 [2].

Although there has been a significant decline in suicide rates between 1990 and 2013 in all European, American (except central Latin America) and the East Asian region, the South Asian region has witnessed a rise [1]. Other studies have reported similar trends in the most populated countries in these regions, India and China [3, 4].

Suicidal behaviour is a complex phenomenon that is influenced by several interacting factors-personal, social, psychological, cultural, biological and environmental. It refers to a range of behaviours that include thinking about suicide (suicidal ideation), planning for suicide, attempting suicide and suicide itself [5]. Those with suicidal ideation, history of self harm or non-fatal suicide attempt are at maximum risk for committing suicide [5]. In order to prevent suicidal deaths, it is essential to identify such individuals with psychosocial crisis and link them to suicide prevention services and support.

Adolescence is a vulnerable stage in one’s life exposed to heightened risks and challenges. Suicide is recognised as the third leading cause of death among adolescents globally [2]. Suicidal behaviours develop during adolescence and peak in late adolescence and early adulthood [6]. Population-based studies indicate that suicidal behaviours in this group share a common risk profile [7]. But most of these studies have been conducted in developed countries with little evidence from the LMICs. Analysis of the Global School Based Student Health Survey (GSHS) conducted across several countries have revealed several risk factors of suicidal behaviours such as poor socioeconomic status, history of bullying, physical attacks, loneliness, anxiety, depressive symptoms, tobacco and alcohol use, sleep disturbances, lack of parental support, few friends and going to bed hungry. Parental engagement, social relationships and close friends were found to be protective factors [8, 9]. Information on the risk and protective factors of suicidal behaviour is essential for planning effective national suicide prevention plan.

In Bhutan, suicide is an important public health concern lately with an alarming rate of over 10 per 100,000 among the general population of all ages in 2014 [10]. The total number of suicide deaths outnumbered deaths due to TB, malaria and HIV combined in 2014 as per the Annual Health Bulletin [11]. Although the global suicide rate has seen a drop, suicide rates in Bhutan have remained steady and rather increased particularly in the last few years [12]. Considering the magnitude and seriousness of the issue, a National Task Force was formed in the Ministry of Health (MoH) to formally establish the National Suicide Prevention Programme in 2014 [12]. Subsequently, the National Suicide Survey was also carried out in 2014 which reported high suicide rates in the 15–40 year age group [10]. The government is committed to tackle these preventable deaths. However, the causes of suicide are complex and multi-factorial.

Adolescents in Bhutan comprise of nearly one-fourth of the total population. They are exposed to the risks of sexual and reproductive health problems, mental disorders, nutritional imbalances, violence and delinquency, increasing use of tobacco and other drugs and social issues like school drop outs and unemployment [13]. A study on the mental health issues among young Bhutanese people showed that nearly half of the adolescents have some mental disorder, most common being depression, anxiety, epilepsy and behavioural disorder due to alcohol or drug use. These factors make them vulnerable to suicidal behaviours [14].

There are limited studies on the prevalence of suicidal behaviour and its associated factors among the school going adolescents in Bhutan.

Thus, the present study was conducted to determine the prevalence and the associated factors (demographic, psycho-social and substance abuse related) of self-reported suicidal behaviour (suicidal ideation and attempt) among school going adolescents aged 13–17 years in Bhutan who were part of the GSHS 2016. As the country is in the process of implementing a National Suicide Prevention Action Plan, the study findings will aid in formulating effective suicide prevention strategies.

## Study methodology

### Study design

This is a cross-sectional analytical study involving secondary analysis of GSHS survey conducted in 2016.

### Study setting

Bhutan is a small mountainous country located in the heart of the Himalayas with an estimated population of around 735,553 according to the latest population census in 2017. About 62.2% of the population lives in the rural areas and 37.8% of the people live in the urban centres. Adolescents (aged between 10 and 19 years) account for 18.9% of the overall population [15]. In 2017, there were 515 schools, of which 479 were public schools and 36 private. The proportion of female to male enrolment in the schools is 1:1.There were nearly 63, 897 students enrolled in Class 7–11 in all schools in Bhutan in 2017 which roughly corresponds to the age group targeted by the GSHS [16].

### Specific setting: GSHS survey 2016

The GSHS was a collaborative project developed by the World Health Organization (WHO) in collaboration with other United Nation organizations; and with technical assistance from Centers for Disease Control and Prevention (CDC), USA. GSHS is a school-based survey conducted primarily among students aged 13–17 years. The survey uses a self-administered questionnaire to obtain data on young people’s health behaviour and protective factors related to the leading causes of morbidity and mortality worldwide [17].

In 2016, a nationally representative survey was conducted among school-going adolescents in Bhutan based on the GSHS methodology. The survey collected information on various dimensions of adolescent health including suicidal behaviours. Adolescents aged 13–17 from 50 selected schools were anonymously self-administered by an 84-item questionnaire, covering demographics and behavioral issues [18].

#### Sample size and sampling

In Bhutan, considering a conservative estimate of 50%, sample size was estimated with a precision of ±5% and found out to be 385. Taking a design effect of 2.5, response rate of 80% and general school attendance of 80%, the sample size was 3125. To present estimates in two subgroups (rural and urban) the sample size was multiplied by two to get the final sample size of 6250.

The GSHS employed a two–stage cluster sample design to produce a nationally representative sample of all students enrolled in classes 7 to 11 (which corresponds to the age group 13–17 years). In the first stage, schools were selected with probability proportional to enrolment size, using a random start. A total of 50 schools were sampled. Equal number of schools were selected from rural (*n* = 25) and urban areas (*n* = 25). A total of 04 private schools were part of the study sample which is similar to the overall proportion of private schools among all schools in Bhutan. In the second stage, classes were selected from each school randomly. In each school, classes were ordered in a list from 7 to 11 and a pre-defined random number sequence was used to select the classes. More the number of classes in a school, more classes were selected based on the random number sequence. All the students in the selected classes were eligible to participate.

A total of 50 schools (25 each in rural and urban) and 7990 students from 210 classes were sampled for inclusion. Of these, all 50 schools and 7578 students completed the questionnaires (7576 questionnaires were found to be valid) [18]. Thus, the overall response rate was 95%. Considering the age range of 13–17 years, a total of 5809 participants were included in this study.

#### Data collection and management

The GSHS was based on a standardized questionnaire which was developed in collaboration with WHO and the CDC that can be administered during one regular class period. The questionnaire was field tested in five conveniently selected non-sampled schools, both in the rural and urban areas, in two districts of Bhutan. Initially, the questionnaire consisted of 90 questions, but after the pilot test, the number of questions was brought down to 84. The questionnaire was self-administered anonymously in English language [18]. A team of data collectors consisting of a survey administrator and a survey coordinator visited each school after taking prior permission from the school authorities for data collection. Before distributing the forms, some of the key terms in the survey were explained to the students and clarification was sought if they understood it well. The operational definitions of the terms used were given in the questionnaire for their better understanding. The school teachers were not present while the students were completing the form. The students were made to sit at a fixed distance from each other and instructed not to talk or discuss questions with each other. If case of any queries, they were told to ask the survey coordinator/administrator who was present there. Following the completion of the questionnaire, weight and height were measured using the standard GSHS methodology with validated instruments (Stadiometer and weighing machine).

The students were asked to fill in the circles of their choice on the answer sheets (optical character recognition [OCR] form). Immediately after the survey was completed, the survey administrators did the necessary cleaning of OCR answer sheets such as, multiple response edits, refilling if the students had not filled the responses properly, other quality checks etc. The data was then were sent to the CDC, where they were scanned and the responses were imported into a database [18].

### Study population

All secondary school going adolescents aged 13–17 years in Bhutan constitute the study population. For the present study, we have analyzed data from adolescents aged 13–17 years who were part of the GSHS study.

### Study variables

The variables extracted from the survey were age, sex, type of student (day scholar and boarding students), schools’ location (urban or rural), body image, physical attack, sexual violence, physical fight, habits such as tobacco abuse, alcohol and drug use, bullying, parental engagement and support, loneliness, food insecurity, lack of sleep, parental smoking and drinking. Variables related to suicidal behaviours included suicidal ideation and suicidal attempt.

Operational definitions of the variables are given in Table 1.

**Table 1:** Operational definitions of data variables according to the Global School-based Student Health Survey 2016 guidelines

Sl. No.	Variables	Definition
1	Suicidal behaviour (ideation and attempt)	Suicidal ideation: During the past 12 months, did you ever seriously consider attempting suicide? (Yes/No) Suicidal attempt: During the past 12 months, how many times did you actually attempt suicide? a)0 times b) 1 time c) 2–3 times d) 4–5 times e) 6 or more times Any attempt was considered as ‘yes’
2	Drinking Alcohol	The GSHS defined drinking alcohol as drinking Ara, Bangchhang, Singchang, Changkoe, beer, whiskey, tongpa, or wine and it did not include drinking a few sips of wine for religious purposes. A “drink” is a glass of wine, a bottle of beer, a small glass of liquor, or a mixed drink Currently drank alcohol is defined as having at least one drink of alcohol on at least one day during the last 30 days
3	Current smoker	Any students smoking cigarettes during the last 30 days. Currently smoking is defined as one who smoked cigarette on at least 1 day during the last 30 days)
4	Current drug use	Any drug use (includes marijuana also called ganja, kayna, black or weed, cocaine, inhalants, SP, N10 or dendrite) during the last 30 days.
5	Bullying	Bullying occurs when a student or group of students say or do bad and unpleasant things to another student. It is also bullying when a student is teased a lot in an unpleasant way or when a student is left out of things on purpose. It is not bullying when two students of about the same strength or power argue or fight or when teasing is done in a friendly and fun way. During the past 30 days, on how many days you were bullied? a)0 days b)1–2 days c)3–5 days d)6–9 days e)10–19 days f)20–29 days g) all days Bullying was defined in this study as bullied on one or more days during the last 30 days
6	Physical attack	A physical attack occurs when one or more people hit or strike someone, or when one or more people hurt another person with a weapon (such as a stick, knife, or gun). It is not a physical attack when two students of about the same strength or power choose to fight each other. During the past 12 months, how many times were you physically attacked? a) 0 times b) 1 time c) 2 or 3 times d) 4 or 5 times e) 6 or 7 times f) 8 or 9 times g) 10 or 11 times h) 12 or more times Defined in this study as physically attacked one or more times during the last 12 months
7	Physical fight	A physical fight occurs when two students of about the same strength or power choose to fight each other. During the past 12 months, how many times were you in a physical fight? a) 0 times b) 1 time c) 2 or 3 times d) 4 or 5 times e) 6 or 7 times f) 8 or 9 times g) 10 or 11 times h) 12 or more times Defined in this study as involved in a physical fight one or more times during the last 12 months
8	Sexual violence	Student forced to have sexual intercourse when he/she did not want to. Have you ever been forced to have sexual intercourse when you did not want to? a) Yes b) No
9	Body image	Self perception of one’s own weight. The following question is asked: How do you describe your weight? a) very underweight b) slightly underweight c) about the right weight d) slightly overweight e) very overweight
10	Urge to use drugs/alcohol	During the past 12 months, how often have you been so worried about something that you wanted to use alcohol or other drugs to feel better? a) never b) rarely c) sometimes d) most of the time e) always Defined in this study as: wanted to use alcohol/other drugs to feel better most of the time or always in the past 12 months
11	Loneliness	During the past 12 months, how often have you felt lonely? a) never b) rarely c) sometimes d) most of the time e) always Defined in this study as: felt lonely most of the time or always in the past 12 months
12	Lack of sleep	During the past 12 months, how often have you been so worried about something that you could not sleep at night? a) never b) rarely c) sometimes d) most of the time e) always Defined in this study as: could not sleep most of the time or always in the past 12 months
13	Other tobacco use	Currently used any tobacco products other than cigarettes (on at least 1 day during the last 30 days)
14	Food insecurity	During the past 30 days, how often did you go hungry because there was not enough food in your home or boarding school? a) never b) rarely c) sometimes d) most of the time e) always Defined in this study as: went hungry most of the time or always because there was not enough food during the last 30 days
15	Close friends	How many close friends do you have? a)0 b) 1 c) 2 d) 3 or more Defined in this study as: having at least one close friend
16	Parental engagement	During the past 30 days, how often did your parents or guardians understand your problems and worries? a) never b) rarely c) sometimes d) most of the time e) always

### Data analysis

Data were imported from the survey database into STATA version 13.1 (Statacorp, College Station, TX, USA) for analysis. The key outcome variables such as suicidal ideation and suicidal attempt were summarized using weighted proportions. Unadjusted prevalence ratios (PRs) and their 95% confidence intervals were calculated to establish the association of socio-demographic and other characteristics with the outcome variables. Log binomial regression analysis was carried out to estimate the adjusted PRs (ENTER method). We also ran a logistic regression model to present the adjusted odds ratios (aORs). Model fit was tested using Likelihood Ratio test and multi-collinearity was also checked to select the appropriate variables to be included in the model. Weighted analysis was carried out. The weights derived were a combination of sampling weight, non-response weight and post-stratification adjustment weight.

### Ethical issues

Ethical approval was sought from the Union Ethics Advisory Group, Paris, France and The Research Ethics Board of Health in Bhutan. As this study involved analysis of secondary data from the GSHS survey conducted in 2016, a waiver of informed consent was obtained.

## Results

A total of 5809 adolescents aged between 13 and 17 years participated in the survey. The demographic, behavioral and psycho-social characteristics are shown in Table 2. More than half of the respondents were females (53.5%, *n* = 3255), day scholars (58.7%, *n* = 3184) and belonged to the urban areas (56.3%, *n* = 3116). One out of every nine adolescents felt that they were overweight (11.4%, *n* = 654); similarly, 2.1 (*n* = 112) students felt they were underweight. More than one-quarter of the adolescents (26.5%, *n* = 1475) had experienced bullying in the last 30 days, 7.1% (*n* = 410) experienced sexual violence before and 38.9% (*n* = 2231) were physically attacked in the past 12 months. About 12.4% (*n* = 721) of the respondents reported loneliness. Nearly one-fourth (24.6%, *n* = 1359) of them were smokers, 29.4% (*n* = 1629) were smokeless tobacco users, and 7.2% (*n* = 385) were using drugs.

**Table 2:** Socio-demographic, behavioural and psycho-social characteristics among school going adolescent aged 13–17 years in Bhutan, 2016

Variables	Frequency (*N* = 5809)	Percentage (%)
Total		
Age group		
13–15 years	3027	(52.8)
16–17 years	2782	(47.2)
Sex		
Male	2515	(46.2)
Female	3255	(53.1)
Missing	39	(0.7)
Type of student		
Day Scholar	3184	(57.3)
Boarding	2474	(40.2)
Missing	151	(2.5)
Location		
Rural	2693	(43.7)
Urban	3116	(56.3)
Underweight		
Yes	112	(2.1)
No	5553	(95.2)
Missing	144	(2.7)
Overweight		
Yes	654	(11.1)
No	5011	(86.2)
Missing	144	(2.7)
Food insecurity		
Never/rare	4036	(69.6)
Sometimes	1588	(27.2)
Most/always	181	(3.1)
Missing	04	(0.08)
Close friend		
None	503	(8.6)
1	889	(15.1)
2	1111	(19.0)
3 or more	3259	(56.5)
Missing	47	(0.8)
Helpful friend	2424	(41.8)
Yes	2424	(41.7)
No	3370	(58.1)
Missing	15	(0.2)
Parents Engagement		
Low	762	(13.1)
Medium	2136	(36.2)
High	2911	(50.7)
Physical Attack		
Yes	2231	(38.9)
No	3568	(60.9)
Missing	10	(0.2)
Physical Fight		
Yes	2306	(40.1)
No	3483	(59.6)
Missing	20	(0.3)
Sexual Violence		
Yes	410	(7.1)
No	5368	(92.4)
Missing	31	(0.5)
Bullying		
Yes	1475	(25.6)
No	4145	(71.0)
Missing	189	(3.4)
Loneliness		
Yes	721	(12.2)
No	5003	(86.3)
Missing	85	(1.5)
Lack of sleep		
Yes	446	(7.6)
No	5356	(92.3)
Missing	07	(0.1)
Urge to use drugs/alcohol		
Never/rare	4774	(82.1)
Sometimes	819	(14.1)
Most/always	202	(3.6)
Missing	14	(0.2)
Current smoker		
Yes	1359	(24.5)
No	4416	(74.9)
Missing	34	(0.6)
Other tobacco use		
Yes	1629	(70.3)
No	4153	(29.2)
Missing	27	(0.5)
Drug abuse		
Yes	385	(7.1)
No	5390	(92.3)
Missing	34	(0.6)
Suicidal Ideation		
Yes	667	(87.2)
No	5072	(11.6)
Missing	70	(1.2)
Suicidal Attempt		
Yes	656	(88.1)
No	5119	(11.3)
Missing	34	(0.6)

Weighted analysis done; Percentages are weighted values

Frequencies are unweighted counts

### Prevalence of self-reported suicidal behaviour

**Fig. 1:**
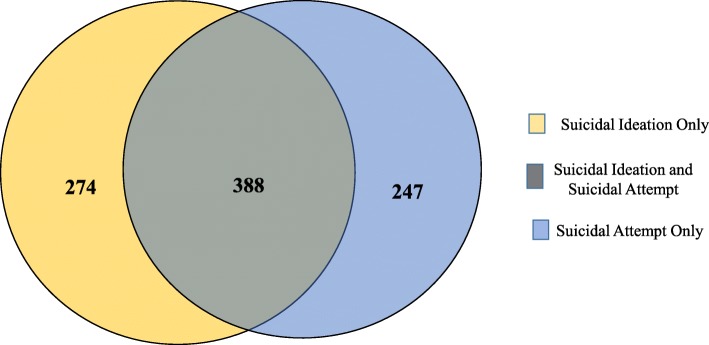
Suicidal ideation, suicidal attempt and their overlap among school-going adolescents in Bhutan 2016. Blue colour indicates suicidal attempt only (*n* = 247). Yellow colour indicates suicidal ideation only (*n* = 274). Green colour indicates both suicidal ideation and suicidal attempt (*n* = 388)

Nearly 11.6% (*n* = 667) of the adolescents reported considering a suicide attempt whereas 11.3% (*n* = 656) reported attempting a suicide in the past 12 months. Table 2 Among those reporting suicidal ideation, 388/662 (58.6%) reported attempting a suicide and 274/662 (41.4%) had ideation alone, whereas, 247/635 (38.9%) reported attempting a suicide without previous ideation Fig. 1.

### Risk factors of self-reported suicidal behaviour

Female sex (aPR = 1.3, 1.1–1.5), food insecurity (aPR = 2.2, 1.9–2.6), physical attack (aPR = 1.4,1.2–1.6), sexual violence (aPR = 1.3, 1.1–1.5), bullying (aPR = 1.6, 1.4–1.9), feeling of loneliness (aPR = 1.4, 1.3–1.7), low parental engagement (aPR = 1.2, 1.0–1.5), reported worry about lack of sleep (aPR = 1.4, 1.2–1.6), urge to use drugs/alcohol (aPR = 1.3, 1.1–1.6), smokeless tobacco use (aPR = 1.4, 1.1–1.9), drug abuse (aPR = 1.5, 1.3–1.7) and parental smoking (aPR = 1.2, 1.1–1.4) were the factors associated with suicidal attempt. Similar factors were also associated with suicidal ideation. Having helpful friends (aPR = 0.8, 0.7–1.0) was found to be protective against suicide ideation Tables 3 and 4.

**Table 3:** Factors associated with suicidal ideation among school going adolescents aged 13–17 years in Bhutan, 2016 (*N* = 5030)

Variables		Total	Suicidal Ideation (unweighted count)	Percentage (%)	PR (95% CI)	aPR (95% CI)	aOR(95% CI)
Total		5809	667	(11.6)			
Age group	13–15 years	3001	338	(11.4)	1	1	1
	16–17 years	2738	329	(11.8)	1.0 (1.0–1.1)	1.0 (0.9–1.1)	1.0 (0.9–1.3)
Sex	Male	2485	242	(9.7)	1	1	1
	Female	3217	420	(13.1)	1.3(1.3–1.4)**	1.4(1.2–1.7)**	1.7 (1.4–2.2)**
Type of student	Day scholar	3146	360	(11.4)	1	1	1
	Boarding	2444	288	(11.8)	1.0(1.0–1.1)	0.8(0.7–0.9)**	0.9 (0.7–1.0)
Location	Rural	2666	294	(11.1)	1	1	1
	Urban	3073	373	(12.2)	1.1(1.1–1.2)**	1.1(0.9–1.2)	1.1 (0.9–1.3)
Underweight	No	5490	638	(11.6)	1	1	1
	Yes	109	7	(6.2)	0.5(0.4–0.7)**	0.4(0.1–1.2)	0.6 (0.3–1.5)
Overweight	No	4954	575	(11.6)	1	1	1
	Yes	645	70	(10.8)	0.9(0.9–1.0)	1.0(0.8–1.2)	1.0 (0.7–1.3)
Food insecurity	Never/rare	3996	399	(10.0)	1	1	1
	Sometimes	1562	223	(14.2)	1.4(1.3–1.5)**	1.2(1.0–1.3)*	1.2 (1.0–1.5)*
	Always	177	45	(25.5)	2.6(2.3–2.8)**	1.3(1.0–1.6)*	1.6 (1.0–2.5)
Physical Attack	No	3534	331	(9.5)	1	1	1
	Yes	2195	334	(14.9)	1.6(1.5–1.6)**	1.2(1.0–1.3)*	1.2 (1.0–1.5)*
Physical Fight	No	3448	312	(9.2)	1	1	1
	Yes	2273	353	(15.2)	1.7(1.6–1.7)**	1.0(0.9–1.2)	1.2 (1.0–1.5)
Sexual Violence	No	5301	558	(10.5)	1	1	1
	Yes	409	102	(24.4)	2.3(2.9–2.5)**	1.5(1.2–1.7)**	1.7 (1.3–2.3)**
Bullying	No	4106	390	(9.5)	1	1	1
	Yes	1448	241	(16.3)	1.7(1.6–1.8)**	1.3(1.1–1.5)**	1.3 (1.0–1.6)*
Close friend	None	498	78	(16.1)	1	1	1
	1	879	108	(11.9)	0.7(0.7–0.8)**	1.0(0.8–1.3)	1.1 (0.7–1.6)
	2	1100	142	(13.0)	0.8(0.7–0.9)**	1.2(1.0–1.5)	1.2 (0.8–1.7)
	3 or more	3220	331	(10.2)	0.6(0.6–0.7)**	0.7(0.6–0.8)*	0.8 (0.7–0.9)*
Helpful friend	No	3328	442	(13.2)	1	1	1
	Yes	2397	225	(9.4)	0.7(0.7–0.8)**	0.8(0.7–1.0)*	0.8(0.7–1.0)*
Parent engagement	High	2880	264	(9.1)	1	1	1
	Medium	2109	294	(13.9)	1.5(1.5–1.6)**	1.2(1.1–1.4)*	1.2 (0.9–1.7)
	Low	750	109	(14.8)	1.6(1.5–1.8)**	1.3(1.1–1.5)*	1.4 (1.1–1.7)**
Loneliness	No	4953	460	(9.3)	1	1	1
	Yes	711	182	(25.2)	2.7(2.6–2.8)**	1.8(1.6–2.2)**	2.1 (1.7–2.7)**
Sleepless Night	No	5295	546	(10.3)	1	1	1
	Yes	438	119	(27.0)	2.6(2.5–2.8)**	1.4(1.1–1.7)*	1.8 (1.4–2.5)**
Urge to use drugs/alcohol	Never/rare	4725	429	(9.1)	1	1	1
	Sometimes	803	166	(21.0)	2.3(2.1–2.4)**	1.5(1.3–1.7)**	1.8 (1.4–2.3)**
	Always	198	65	(31.0)	3.4(3.1–3.7)**	1.4(1.1–1.8)*	2.1 (1.4–3.2)**
Smoking	No	4368	427	(9.7)	1	1	1
	Yes	1339	231	(17.2)	1.8(1.7–1.9)**	1.3(1.0–1.8)	1.4 (0.8–2.2)
Other Tobacco use	No	4109	394	(9.6)	1	1	1
	Yes	1604	267	(16.7)	1.7(1.6–1.8)**	0.8(0.6–1.2)	0.9 (0.6–1.5)
Drugs Abuse	No	5327	577	(10.7)	1	1	1
	Yes	378	85	(22.2)	2.1(1.9–2.2)	1.5(1.3–1.9)**	1.3 (0.9–1.9)
Parental smoking	No	3610	386	(11.0)	1	1	1
	Yes	2108	275	(13.0)	1.2(1.1–1.3)**	1.2(1.1–1.4)*	1.2 (1.0–1.4)
Parental drinking	No	2672	299	(11.3)	1	1	1
	Yes	3031	360	(12.0)	1.0(1.0–1.1)	1.0(0.8–1.1)	1.0 (0.8–1.2)

*PR* Prevalence Ratio, *aPR* Adjusted Prevalence Ratio

*aOR* Adjusted Odds ratio

*p-value< 0.05; ** *p*-value< 0.001

Weighted analysis done

1 stands for Reference category

**Table 4:** Factors associated with suicidal attempt among school going adolescents aged 13–17 years in Bhutan- 2016 (*N* = 5053)

Variables		Total	Suicidal attempt (unweighted count)	Percentage (%)	PR (95% CI)	aPR (95% CI)	aOR (95% CI)
Total		5809	656	(11.3)			
Age group	13–15 years	3009	322	(10.7)	1	1	1
	16–17 years	2766	334	(11.9)	1.1 (1.1–1.2)**	1.0 (0.9–1.2)	1.0 (0.8–1.2)
Sex	Male	3236	392	(10.3)	1	1	1
	Female	2500	258	(12.1)	1.2 (1.1–1.2)**	1.3 (1.1–1.5)**	1.6 (1.3–2.0)
Type of student	Day scholar	3165	326	(10.1)	1	1	1
	Boarding	2462	311	(12.9)	1.3 (1.2–1.3)**	0.9 (0.8–1.0)	1.0 (0.8–1.2)
Location	Rural	2676	267	(10.1)	1	1	1
	Urban	3099	389	(12.7)	1.2 (1.2–1.3)**	1.1 (0.9–1.2)	1.2 (1.0–1.5)*
Underweight	No	5521	633	(11.4)	1	1	1
	Yes	112	6	(4.8)	0.4 (0.3–0.6)**	0.4 (0.1–1.2)	0.4 (0.1–1.2)
Overweight	No	4984	569	(11.4)	1	1	1
	Yes	649	70	(10.6)	0.9 (0.9–1.0)	0.9 (0.7–1.1)	1.0 (0.7–1.4)
Food insecurity	Never/rare	4016	357	(8.7)	1	1	1
	Sometimes	1575	236	(15.0)	1.7 (1.6–1.8)**	1.2 (1.1–1.4)*	1.3 (1.0–1.6)*
	Always	180	62	(34.7)	4.0 (3.7–4.3)**	2.2 (1.9–2.6)**	3.7 (2.4–5.6)**
Physical Attack	No	3551	272	(7.6)	1	1	1
	Yes	2217	383	(17.0)	2.2 (2.1–2.4)**	1.4 (1.2–1.6)**	1.7 (1.4–2.1)**
Physical Fight	No	3467	272	(7.8)	1	1	1
	Yes	2291	380	(16.4)	2.1 (2.0–2.2)**	1.1 (0.9–1.3)	1.2 (1.0–1.5)
Sexual Violence	No	5340	546	(10.1)	1	1	1
	Yes	406	102	(25.4)	2.5(2.3–2.7)**	1.3(1.1–1.5)*	1.6 (1.2–2.2)*
Bullying	No	4128	331	(7.9)	1	1	1
	Yes	1461	277	(18.8)	2.4 (2.2–2.5)**	1.6 (1.4–1.9)**	1.7 (1.3–2.0)**
Close friend	None	493	80	(16.3)	1	1	1
	1	886	107	(12.0)	0.7 (0.7–0.8)**	0.9 (0.8–1.2)	0.8 (0.6–1.2)
	2	1106	142	(13.0)	0.8 (0.7–0.9)**	1.1 (0.9–1.3)	0.9 (0.6–1.3)
	3 or more	3250	313	(9.4)	0.6 (0.5–0.6)**	0.9 (0.7–1.0)	0.7 (0.5–1.0)*
Helpful friend	No	3346	430	(12.5)	1	1	1
	Yes	2414	223	(9.4)	0.7 (0.7–0.8)**	1.0 (0.9–1.3)	0.9 (0.7–1.1)
Parent engagement	High	2891	256	(8.7)	1	1	1
	Medium	2128	280	(13.1)	1.5 (1.4–1.6)**	1.2 (1.0–1.3)**	1.5 (1.1–2.0)**
	Low	756	120	(16.0)	1.8 (1.7–2.0)**	1.2 (1.0–1.5)*	1.3 (1.1–1.6)*
Loneliness	No	4975	467	(9.4)	1	1	1
	Yes	719	176	(24.1)	2.6 (2.4–2.7)**	1.4 (1.3–1.7)**	1.6 (1.2–2.1)**
Sleepless Night	No	5328	536	(10.0)	1	1	1
	Yes	440	118	(26.8)	2.7(2.5–2.9)**	1.4(1.2–1.6)**	1.8 (1.3–2.5)**
Urge to use drugs/alcohol	Never/rare	4748	418	(8.7)	1	1	1
	Sometimes	813	172	(21.0)	2.4 (2.3–2.5)**	1.4 (1.2–1.6)**	1.7 (1.3–2.2)**
	Always	200	63	(31.0)	3.5 (3.3–3.8)**	1.3 (1.1–1.6)*	1.7 (1.1–2.7)*
Smoking	No	4389	405	(9.1)	1	1	1
	Yes	1354	241	(17.4)	1.9 (1.8–2.0)**	0.9 (0.7–1.1)	0.9 (0.6–1.4)
Other Tobacco use	No	4126	362	(8.6)	1	1	1
	Yes	1624	289	(17.5)	2.0 (1.9–2.1)**	1.4 (1.1–1.9)*	1.6 (1.1–2.5)*
Drugs Abuse	No	5360	550	(10.1)	1	1	1
	Yes	381	98	(25.5)	2.5 (2.4–2.7)**	1.5 (1.3–1.7)**	1.6 (1.1–2.3)*
Parent smoking	No	3629	366	(10.0)	1	1	1
	Yes	2126	286	(13.4)	1.3 (1.3–1.4)**	1.2 (1.1–1.4)*	1.3 (1.1–1.7)*
Parent drinking	No	2681	295	(11.0)	1	1	1
	Yes	3057	354	(11.4)	1.0 (1.0–1.1)	0.9 (0.8–1.0)	0.9 (0.7–1.1)

*PR* Prevalence Ratio, *aPR* Adjusted Prevalence Ratio

*aOR* Adjusted Odds ratio

*p-value< 0.05; ** *p* value< 0.001

Weighted analysis done

1 stands for Reference category

## Discussion

One out of every nine adolescents aged 13–17 years in Bhutan reported attempting a suicide in the past 12 months. The high suicidal rate among adolescents is a matter of grave concern, especially for a country like Bhutan. Female gender, food insecurity, physical attack, bullying, sexual violence, loneliness, lack of sleep, drug abuse and parental smoking were identified as the key risk factors.

In a pooled analysis of the GSHS survey across 59 LMICs in six regions of WHO, the prevalence of suicidal ideation and attempt was found to be 16.9 and 17.0% respectively, highest in the African region and lowest in the Southeast Asia region [19]. The prevalence of self-reported suicidal ideation and attempt in this age group ranged from 4.9–13.7% and 3.9–13.3% respectively in the Asian region [20]. These estimates (suicidal ideation vs suicidal attempt) were higher in Bhutan along with Thailand (12.5% vs 13.3%), Maldives (13.1% vs 12.7%) and Nepal (13.6% vs 10.3%) compared to other South-East Asian countries like Bangladesh (4.9% vs 6.7%), Srilanka (9.4% vs 6.8%), Timor-Leste (9.3% vs 9.5%), Myanmar (9.4% vs 8.8%) and Indonesia (5.4% vs 3.9%) where the proportions are relatively lower [20, 21]. Suicidal ideation and attempt rate seem to differ across countries. This is probably because suicidal behaviour has a large number of underlying factors at individual, community and societal level that are complex and interactive.

Physical attack and bullying has been shown to be a strong risk factor for self-reported suicidal ideation and attempt among adolescents. This has been reported unequivocally in previous literature from different countries. This relationship is often mediated by other factors [22]. Those who are bullied are more likely to be depressed or anxious [23], have lower academic achievement, report feeling like they do not belong at school [24], have poorer social and emotional adjustment, greater difficulty making friends, have poorer relationships with classmates and experience loneliness [25].

Female gender has been found to be strongly associated with self-reported suicidal behaviours compared to males which is supported by a large body of evidence across all age groups [26]. Men and women differ in their roles, responsibilities, status and power in the society. Females are more emotionally labile and exposed to different hardships in life. Previous studies have shown that females are at a higher risk of being sexually abused than their male counterpart [20, 27]. It is believed that these socially constructed differences interact with biological differences to contribute to this gender association [28].

The present study reported a link between parental smoking and self-reported suicidal behaviour among adolescents. Previous study analyzing a large national youth survey data in the United States of America reported that those with a parent who smoke do not have a statistically significant increased risk of suicidal ideation regardless of teen smoking behaviour [29]. In light of this limited contrasting evidence, it is difficult to make any conclusion now, thus warranting further research.

Feeling of loneliness has been proven to be a strong predictor of suicidal behaviour in several cross-sectional and longitudinal studies, similar to the present study [30, 31]. A recent meta-analysis found that people experiencing loneliness were twice more likely to have suicidal ideation and suicide [32]. A content analysis of online suicide notes found loneliness as one of the core themes [33]. Loneliness is due to lack of desired social relationships and have been linked to various correlates of suicidal behaviour such as depression, anxiety and other mental health problems and substance use [34–36].

This is further supported by another finding of our study that having 3 or more close friends had protective effect against suicidal ideation and attempt. Other studies from Nepal and China had also revealed similar associations between having close friends and suicidal behaviours [37, 38]. Studies have also suggested that feeling of loneliness is likely to play a role in the association between adolescent peer relationships and suicide ideation and attempts [37, 39]. This reinforces the importance of peer support in schools in maintaining mental well-being.

Lack of sleep has also been shown to be associated with the risk of self-reported suicidal behaviours which finds concurrence in several other studies [40, 41]. The diathesis stress model has been put forwarded to explain this association and also the role of sleep disturbances in several other mental health problems such as depression and substance abuse which is believed to mediate suicidal behaviour [40].

Those found to use drugs or tobacco were more likely to have suicidal tendencies [42]. Substance use among adolescents in Bhutan was higher than most other countries in the SEAR [20]. This has serious implications not only on the mental health, but also physical health and the future of these young people. The issue of drug abuse among adolescents should not be neglected while implementing suicide prevention plans.

There were a few strengths in this study. First, data were obtained from a national representative survey which follows a globally recognized standard GSHS methodology with a high response rate. This makes the study findings generalizable to the adolescent population aged 13–17 years in the country. Second, the subject under study i.e. suicidal behaviour among adolescents is an identified research priority in the country. Third, data quality was ensured though standard data cleaning and management procedures at various levels supported by the CDC, Atlanta. Fourth, weighted analysis was carried out taking into account sampling weight, non-response weight and post-stratification adjustment weight.

However, the study had some limitations as well. The major limitation in this study was that the assessment of outcome and other key variables was based on self-report by the participant with no means of validating the responses. There are chances that some of the adolescents might not have fully comprehended some of the questions and this may have affected their response. However, a trained survey administrator was always present during the survey to provide any clarification. The questionnaire also had detailed explanations of different key variables. The survey questionnaire was self-administered in English language which questions the reliability and validity of the responses. This is because despite English being the medium of education in schools, it is not their local/native language. However, all efforts were made to ensure that the students understood the questions well. The key terms in the survey were explained to the students and clarification was sought if they understood it well. The school teachers and survey administrator were not present while the students were completing the form to clarify any doubts.

The survey did not capture information on those students who died as a result of committing suicide, were school drop outs or who never went to a school or those who were not present in school on the day of the survey. The survey also excluded young monks who reside in monastic institutions. The stigma surrounding suicidal behaviours in Bhutanese societies might have caused an underreporting of the conditions due to social desirability bias. These limitations are likely to underestimate the study results.

The study results have important policy implications. First, the alarming rates of suicidal behaviour among school-going adolescents call for a structured school-based intervention to identify and tackle the risk factors of suicide. The National Suicide Action Plan also bats for suicide prevention in schools [12]. School personnel, especially teachers and parents need professional training and support to help them build the skills and confidence to identify and assist vulnerable youth. Schools also need to take a greater role in handling bullying or any physical attack and drug abuse as it occurs largely within the school’s premises. The ministry has already taken a positive step in this regard by appointing a counselor in each school. However, the roles of these counselors in prevention and management of suicides have not been clearly defined. This study has identified key areas of intervention where the counselors can play a major role.

Second, loneliness could be an important focus of preventive action. Reducing loneliness is one of the most direct ways of tackling suicidal behaviour along with other mental health challenges, including depression, hopelessness, anxiety and substance abuse. We should create platforms for adolescents to share their stories, burden of pain, joy and despair. More outdoor activities should be encouraged to forge a sense of connection and belonging. Use of mobile phones has been linked to loneliness which also needs to be taken care of by parents and teachers [43].

Third, parental engagement was found to be a protective factor against self-reported suicidal behaviour concurring with the findings in other countries of the region [20]. It was also found protective against other mental health problems and substance use. Thus, parents can play an important role in early identification of these risk factors and mitigating them. Parents should create such environment at home so that children discuss everything with them freely.

## Conclusion

There is high prevalence of self-reported suicidal behaviours among school going adolescents in Bhutan, more among girls. Bullying, sexual violence, feeling of loneliness and drug abuse were the key risk factors identified. It is important to identify these risk factors early and effectively tackle them in order to prevent suicides. It requires a multi-faceted intervention with the support of the children, community, teachers and parents.

## Data Availability

GSHS survey was a national survey conducted by the Ministry of Health, Bhutan in collaboration with the CDC, USA and the WHO. The survey dataset is available with the Ministry of Health, Bhutan and might be produced on request after taking necessary approval.

## Abbreviations

CDC: Centers for Disease Control and Prevention
DALYs: Disability Adjusted Life Years
GSHS: Global School Based Student Health Survey
LMICs: Low-Middle-Income Countries
OCR: Optical Character Recognition
WHO: World Health Organization
